# Coexisting sublingual dermoid cyst and heterotopic gastrointestinal cyst: Case report

**DOI:** 10.4317/jced.53817

**Published:** 2018-02-01

**Authors:** Göksel Şimşek-Kaya, İrem-Hicran Özbudak, Dinçer Kader

**Affiliations:** 1 Associate Professor, Department of Oral and Maxillofacial Surgery, Faculty of Dentistry, Akdeniz University, Antalya, Turkey; 2 Associate Professor, Department of Pathology, School of Medicine, Akdeniz University, Antalya, Turkey; 3 Research Assistant, Department of Oral and Maxillofacial Surgery, Faculty of Dentistry, Akdeniz University, Antalya, Turkey

## Abstract

A heterotopic oral gastrointestinal cyst coexisting with a lingual dermoid cyst is a rare condition, with only 3 reported cases in the English-language literature. Clinically, both gastrointestinal and dermoid cysts tend to manifest as an asymptomatic swelling. Cysts above the mylohyoid often present with sublingual swelling, whereas those below the mylohyoid present with submental swelling. Increased salivation, altered speech, dyspnea and difficulties in eating, swallowing and respiration may be present, depending on the size of the cyst. This paper describes the case of a 4-year-old girl presenting with swelling of the tongue and floor of the mouth that interfered with normal speech. Two cystic lesions were surgically excised using an intraoral approach, and recovery was uneventful. Histopathological examination of the specimens taken during surgery revealed the first, anterior cyst to be a dermoid cyst and the second, posterior cyst to be a heterotopic oral gastrointestinal cyst (HGIC). Although this situation is very rare, it should be included in the differential diagnosis of swellings in the submandibular region in the pediatric population.

** Key words:**Dermoid cyst, heterotopic gastrointestinal cyst, children, tongue.

## Introduction

The term ‘heterotopia’, also referred to as ‘choristoma’ or ‘aberrant rest’, is used to describe tissue or organs displaced to an abnormal location (1). A heterotopic gastrointestinal cyst of the oral cavity – also known as a ‘lingual foregut duplication cyst’, ‘enterocystoma’, ‘choriotomatic cyst’, ‘ciliated epithelial cyst’, or ‘heterotopic large bowel cyst’(2), has been observed in the duodenum, gallbladder, jejunum, pancreas, ileum, rectum, anus and the umbilicus as well as in the lips, larynx, lung, submandibular glands, epiglottis and anterior neck (1).

Dermoid cysts are rare, benign, developmental lesions that occur primarily in the testes and ovaries, although they can be found anywhere in the body at an embryological fusion point, including the head and neck areas. Approximately 7% of all dermoid cysts form in the head and neck region, and of those, 23% develop in the ﬂoor of the mouth (3-6). However, dermoid cysts represent only 0.01% of all oral lesions (7). When dermoid cysts do occur in the oral cavity, they are usually found on the floor of the mouth in close association with the mylohyoid muscle, or, rarely, in the tongue (6). If a dermoid cyst is left untreated for a long period of time, carcinomatous change is possible, although this is very rare (8).

Clinically, both gastrointestinal and dermoid cysts tend to manifest as an asymptomatic swelling. Cysts above the mylohyoid often present with sublingual swelling, whereas those below the mylohyoid present with submental swelling. Increased salivation, altered speech, dyspnea and difficulties in eating, swallowing and respiration may be present, depending on the size of the cyst (2,4,6,9). Dyspnea and respiratory distress can also be present, and tracheostomy may occasionally be required (9).

The coexistence of a dermoid cyst and heterotopic oral gastrointestinal cyst (HGIC) is extremely rare. To date, only three cases have been reported in the English-language literature. The following report describes such a case, including the pathogenesis (10).

## Case Report

**Figure 1:**
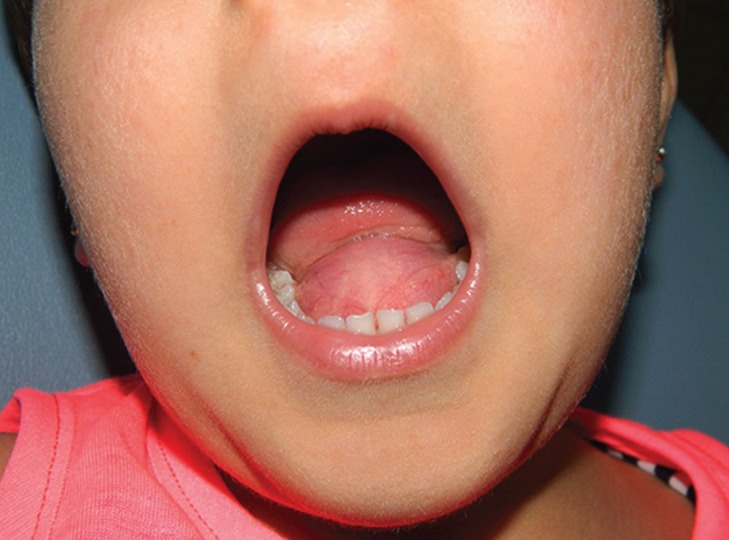
The patient’s preoperative clinical appearance.

**Figure 2:**
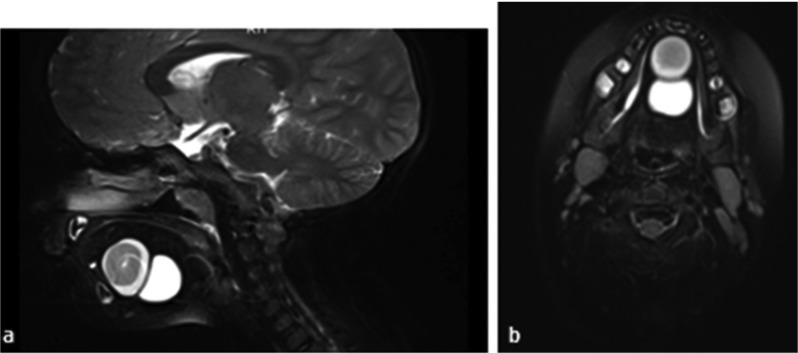
a: Sagittal (Magnetic resonance imaging) MRI scan showing the relationship between the two lesions in the tongue and on the ﬂoor of the mouth. b: Coronal Magnetic resonance imaging (MRI) scanned image of the two lesions.

A 4-year-old girl presented at the Akdeniz University Dental Faculty Department of Oral and Maxillofacial Surgery with a painless sublingual swelling. Extraoral examination revealed no facial abnormalities or swellings. Intraoral examination revealed a large, compressible cystic mass in the midline of the anterior floor of the mouth, causing significant elevation of the tongue. The mucosa covering the mass was normal in color, while the mass itself was smooth, firm and ﬂuctuant at bi-manual palpation, with no evidence of inflammation (Fig. 1). The patient’s parents were uncertain how long the swelling had been present, but stated that their child’s speech was below the level of her peers. The child’s medical history was unremarkable. No regional lymphadenopathy was noted. Magnetic resonance imaging (MRI) revealed contiguous, round, T2-hyperintense cysts with regular margins in the central and anterior parts of the floor of the mouth that were 22x22 mm and 25x21 mm, respectively, with T1-hyperintense areas observed in the anterior cystic lesion (Figs. 2a, b). Preoperative differential diagnoses included epidermoid or dermoid cysts, cystic hygroma, ranula and lymphangioma.

**Figure 3:**
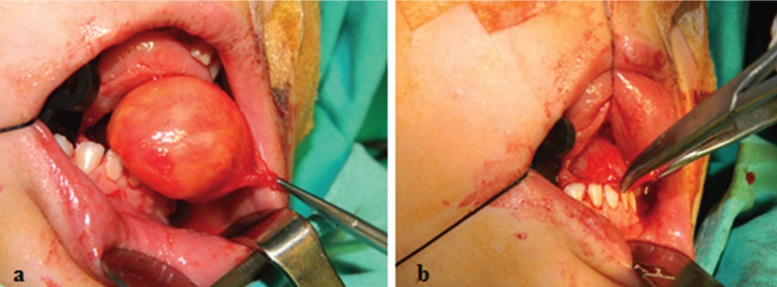
a: Intraoperative appearance of the dermoid cyst. b: Intraoperative appearance of the heterotopic gastrointestinal cyst.

**Figure 4:**
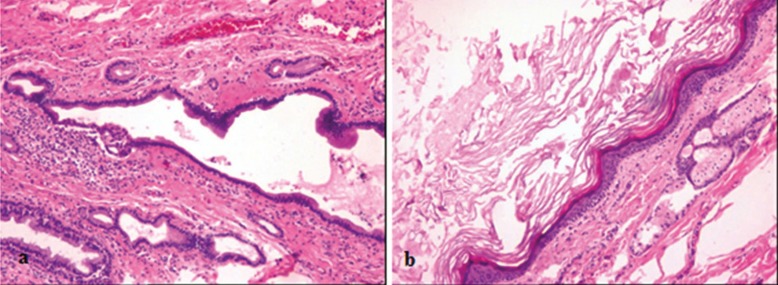
a: Heterotopic gastrointestinal cyst. The part shown consisted of gastrointestinal epithelium. The cystic lining cells included gastrointestinal cells such as mucus, parietal or chief cells (hematoxylin and eosin) (H&E stain) ×100. b: Dermoid cyst. The cyst is lined by a stratified squamous epithelium with a granular layer, and the cyst-filled space contains laminated keratin (hematoxylin and eosin) (H&E stain) ×100.

Under nasotracheal intubation, using an intraoral approach, a semi-lunar incision was made on the ventral surface of the tongue from left to right. Using blunt dissection, the first lesion was separated from the surrounding tissue, and the genioglossus muscle was separated from the lesion. The first lesion was then gently elevated from the mylohyoid muscle and removed in one intact piece (Fig. 3a). Next, the second lesion was accessed through a blunt dissection in the muscles of the floor of the mouth (Fig. 3b) and excised in the same manner as the first lesion. Hemostasis was established, the wound edges were closed, and a 0.25 inch Penrose drain was inserted. The drain was removed on the postoperative 3rd day, and the region was closed primarily via suture. Histopathological examination of the specimens taken during surgery revealed the first, anterior cyst to be a dermoid cyst and the second, posterior cyst to be a heterotopic oral gastrointestinal cyst (HGIC) (Figs. 4a. b). Postoperative healing was uneventful. In line with clinical protocols, the patient presented for regular follow-up visits.

## Discussion

Differential diagnosis of swellings on the floor of the mouth should include cystic lesions such as mucocele, ranula, thyroglossal duct cysts, lymphoepithelial cysts, cystic teratoma, salivary gland tumors and mesenchymal tumors as well as sialadenitis, hemangioma/lymphangioma and abscesses (3-6). The diagnostic imaging for these types of lesions includes computed tomography (CT), ultrasonography (US) and MRI (3,4). Definitive diagnosis depends on histological investigation following clinical and radiological examination (4).

The term ‘dermoid’ is used as a general term to refer to all three histological types of dermoid cysts identified by Meyer (11) as well as to one of the specific histological categories: epidermoid, dermoid and teratoid. Epidermoid cysts have only a stratified squamous epithelial lining with no adnexal structures. In addition to a typical, squamous epithelial lining, dermoid cysts also exhibit dermal appendages, like sebaceous glands, hair follicles and sweat glands. Teratoid cysts have both squamous and respiratory epithelium, dermal appendages and distinct mesodermal components (11).

Surgical excision is the treatment of choice for both HGIC and dermoid cysts. Depending upon the clinical relationship between the cyst and the mylohyoid and geniohyoid muscles, either intraoral or extraoral methods, or sometimes even a combined approach, may be appropriate (3,4,9,12). Prognosis is generally good, with no reports of recurrence after complete surgical excision (3). However, epithelial tracts leading to the mandible or hyoid bone have been documented and require excision to avoid recurrence (12).

In conclusion, although coexisting sublingual dermoid and heterotopic gastrointestinal cysts are very rare, they should be especially included in the differential diagnosis of swellings in the submandibular region in the pediatric population.

## References

[1] Wetmore RF, Bartlett SP, Papsin B, Todd NW (2002). Heterotopic gastric mucosa of the oral cavity: a rare entity. Int J Pediatr Otorhinolaryngol.

[2] Said-Al-Naief N, Fantasia JE, Sciubba JJ, Ruggiero S, Sachs S (1999). Heterotopic oral gastrointestinal cyst: report of 2 cases and review of the literature. Oral Surg Oral Med Oral Pathol Oral Radiol Endod.

[3] Kim IK, Kwak HJ, Choi J, Han JY, Park SW (2006). Coexisting sublingual and submental dermoid cysts in an infant. Oral Surg Oral Med Oral Pathol Oral Radiol Endod.

[4] Pan M, Nakamura YC, Clark M, Eisig S (2011). Intraoral dermoid cyst in an infant: a case report. J Oral Maxillofac Surg.

[5] Ho MW, Crean SJ (2003). Simultaneous occurrence of sublingual dermoid cyst and oral alimentary tract cyst in an infant: a case report and review of the literature. Int J of Paediatr Dent.

[6] Dimtsas S, Theologie-Lygidakis N, Iatrou I (2010). Intralingual dermoid cyst in an infant presenting swallowing and sleeping difficulties. J Clin Pediatr Dent.

[7] Papadogeorgakis N, Kalfarentzos E, Vourlakou C, Alexandridis C (2009). Surgical management of a large median dermoid cyst of the neck caus-ing airway obstruction. A case report. Oral Maxillofac Surg.

[8] El-Hakim IE, Alyamani A (2008). Alternative surgical approaches for excision of dermoid cyst of the ﬂoor of mouth. Int J Oral Maxillofac Surg.

[9] Gordon PE, Faquin WC, Lahey E, Kaban LB (2013). Floor-of-mouth dermoid cysts: report of 3 variants and a suggested change in terminology. J Oral Maxillofac Surg.

[10] Crivelini MM, Soubhia AM, Biazolla ER, Neto SC (2001). Heterotopic gastrointestinal cyst partially lined with dermoid cyst epithelium. Oral Surg Oral Med Oral Pathol Oral Radiol Endod.

[11] Meyer I (1955). Dermoid cysts of the ﬂoor of the mouth. Oral Surg Oral Med Oral Pathol.

[12] Eppley BL, Bell MJ, Sclaroff A (1985). Simultaneous occurrence of dermoid and heterotopic intestinal cysts in the floor of the mouth of a newborn. J Oral Maxillofac Surg.

